# Retraction: Al-Salahi, R.A. *et al*. Cytotoxicity and Anti-Inflammatory Activity of ethylsulfanyltriazoloquinazolines. *Molecules* 2013, *18*, 1434-1446

**DOI:** 10.3390/molecules180911001

**Published:** 2013-09-09

**Authors:** Shu-Kun Lin

**Affiliations:** *Publisher* of *Molecules*, Multidisciplinary Digital Publishing Institute (MDPI), Klybeckstrasse 64, Basel CH-4057, Switzerland; E-Mail: lin@mdpi.com

We have been made aware of the fact that a large proportion of the Introduction section and corresponding references of the title paper [1] had been copied verbatim from an earlier paper by Hamdy and Gamal-Eldeen [2], and further, during our investigation it has also come to light that most of the text in question had been lifted unchanged from an even earlier review paper by a different group [3]. The authors have been contacted, these facts confirmed, and Dr. Amira M. Gamal-Eldeen has been identified as the person responsible for contributing that part of the paper. 

As a member of the Committee on Publication Ethics (COPE), MPDI takes very seriously the responsibility to enforce a rigorous peer-review process together with strict ethical policies and standards to ensure the addition of high quality scientific works to the field of scholarly publication. In addition to an infringement of the Elsevier and American Association for Cancer Research copyright on the previous publications, this is also a clear violation of our policy to only publish new, previously unpublished material, so this paper is declared retracted and shall be marked accordingly for the scientific record. We cannot comment on any of the scientific data contained in the *Molecules* paper, which to the best of our knowledge is original.

We would like to apologize to our readership on behalf of the *Molecules* editorial team for the fact this event went undetected during the peer-review and pre-publication processing of the paper and for any inconvenience caused by this event.
